# mzStudio: A Dynamic Digital Canvas for User-Driven Interrogation of Mass Spectrometry Data

**DOI:** 10.3390/proteomes5030020

**Published:** 2017-08-01

**Authors:** Scott B. Ficarro, William M. Alexander, Jarrod A. Marto

**Affiliations:** 1 Department of Cancer Biology and Blais Proteomics Center, Dana-Farber Cancer Institute, 450 Brookline Avenue, Boston, MA 02115, USA; scott_ficarro@dfci.harvard.edu (S.B.F.); Williamm_alexander@dfci.harvard.edu (W.M.A.); 2 Department of Biological Chemistry and Molecular Pharmacology, Harvard Medical School, Boston, MA 02215, USA; 3 Department of Oncologic Pathology, Dana-Farber Cancer Institute, Boston, MA 02215, USA; 4 Department of Pathology, Brigham and Women’s Hospital, Harvard Medical School, Boston, MA 02215, USA

**Keywords:** bioinformatics software, mass spectrometry, quantification, results distribution, API, application programming interface, SQLite

## Abstract

Although not yet truly ‘comprehensive’, modern mass spectrometry-based experiments can generate quantitative data for a meaningful fraction of the human proteome. Importantly for large-scale protein expression analysis, robust data pipelines are in place for identification of un-modified peptide sequences and aggregation of these data to protein-level quantification. However, interoperable software tools that enable scientists to computationally explore and document novel hypotheses for peptide sequence, modification status, or fragmentation behavior are not well-developed. Here, we introduce mzStudio, an open-source Python module built on our multiplierz project. This desktop application provides a highly-interactive graphical user interface (GUI) through which scientists can examine and annotate spectral features, re-search existing PSMs to test different modifications or new spectral matching algorithms, share results with colleagues, integrate other domain-specific software tools, and finally create publication-quality graphics. mzStudio leverages our common application programming interface (mzAPI) for access to native data files from multiple instrument platforms, including ion trap, quadrupole time-of-flight, Orbitrap, matrix-assisted laser desorption ionization, and triple quadrupole mass spectrometers and is compatible with several popular search engines including Mascot, Proteome Discoverer, X!Tandem, and Comet. The mzStudio toolkit enables researchers to create a digital provenance of data analytics and other evidence that support specific peptide sequence assignments.

## 1. Introduction

Adaptation of false-discovery statistics and peptide-to-protein parsimony rules enable straightforward compilation of large-scale mass spectrometry experiments to a simple list of peptides, proteins, and associated quantification values. While some details will continue to evolve, the field has undoubtedly reached a point where the expression of a large number of proteins can be confidently measured in many biological systems based on assignment of unmodified tryptic peptide sequences and their parsimonious mapping to protein groups or other identifiers. Indeed, this approach provides a global view of the proteome and can reveal how constituent components may respond to biological perturbation. These effects can be visualized with simple heat-map graphics, and the underlying lists of quantified proteins can be distributed in standard spreadsheet files. However, this approach fails to capture the granularity in protein modifications which result from the rich and dynamic chemical environment associated with endogenous physiology. Even in the post-genomic era, new post-translational modifications of proteins have been discovered [1,2,3]. Interrogating mass spectrometry data at this level of functional resolution requires a dynamic and interactive visualization framework on which researchers can experiment with novel hypotheses for peptide sequences and associated modifications. 

In the last several years, many useful tools have been developed for the analysis of proteomic data [4,5]. These tools are typically developed in a task-specific manner. For example, MaxQuant [6] provides for feature detection, database search, and relative quantification, while Skyline [7] focuses on building and refining targeted mass spectrometry assays. Proteowizard [8] provides several tools to convert mass spectrometry data to common file formats (i.e., mzML) and supports basic data display. Other groups have developed databases intended to serve as warehouses for long-term archiving, compilation, and access to MS/MS spectra [9,10]. More recent tools such as Mass++ [11] and Batmass [12] focus on data visualization. Inspired by these efforts, we developed mzStudio, an open-source, Python-based digital canvas for interactive exploration and interpretation of mass spectrometry data. mzStudio is built on our multiplierz framework [13,14,15] and leverages our common API [16] to facilitate user-directed navigation across proprietary native mass spectrometry files and scan types. mzStudio also provides unique capabilities which enable users to build and integrate evidence for novel hypotheses related to specific spectra. First, users can interact directly with search engines (Mascot, X!Tandem, Comet) to iteratively test sequence and modification assignments, or explore unexpected fragmentation behavior. In addition, mzStudio provides on-board spectral processing and feature analysis tools. Finally, mzStudio includes an embedded ‘spectral notebook’, which captures the details and logic that underlie evolving ideas and workflows. With these features, mzStudio expands beyond a simple visualization platform to provide a seamless link between computational interrogation of mass spectra, digital provenance, and publication or other dissemination of results.

## 2. Materials and Methods

### 2.1. Architecture

mzStudio was developed in Python, an easy to understand scripting language that supports rapid prototyping, and is currently deployable from 64-bit Python 2.7. The GUI is implemented with the wxPython 3.0 agw docking library which allows easy window management. A key component of mzStudio is the multiplierz project [13] (version 2.0 [15]), which provides libraries for raw data file access (mzAPI [16]), reading and writing spreadsheets and databases (mzResult [14]), and launching database searches (mzSearch [15]). Additional routines for interrogating mass spectra are accessible via the multiplierz mzTools module [15]. mzStudio and multiplierz are both available under a GPL license. mzStudio source code, as well as a tutorial document, can be downloaded from Github: https://github.com/BlaisProteomics/mzStudio. Example data and search result files are provided on sourceforge: https://sourceforge.net/projects/mzstudio-tutorial-package.

### 2.2. Results

**Figure 1:**
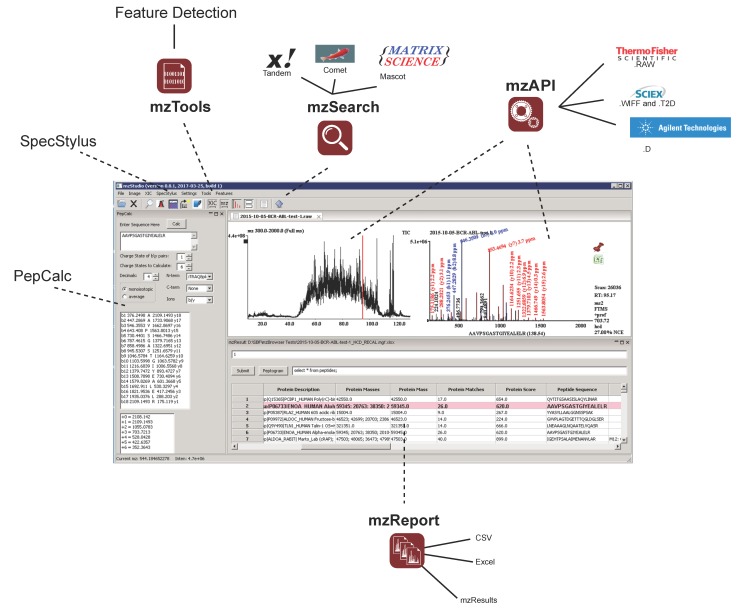
The main user interface of mzStudio supports direct access to native mass spectrometry data files from different instrument manufacturers, and can import database search results from Mascot, X!Tandem, Comet, and Proteome Discoverer. An interactive analysis window enables rapid SQLite filtering of data, while a peptide calculator toolbar displays theoretical fragment ion masses. Additional tools provide for feature detection, custom spectral processing, and launching database searches.

mzStudio was developed in our lab to provide a centralized framework to interactively visualize, annotate, and integrate sequence assignment and other features of mass spectrometry data across instrument manufacturers, platforms, and search engines (Figure 1). Consistent with our design philosophy for our broader multiplierz project, mzStudio provides direct access to native mass spectrometry data files without the need for conversion to auxiliary file formats (i.e., xml); all supported vendors and instrument platforms are listed in Supplementary Table S1. Exemplary file access times are listed in Supplementary Table S2. mzStudio leverages our common API [16] and manufacturer DLLs (installed with multiplierz) to directly access native data files; as such, mzStudio is currently limited to use on Windows OS. mzStudio supports access to and visualization of MS1, MSn, DIA, and specialized triple quadrupole scans (precursor/neutral loss scanning data). mzStudio can currently read SRM data from LTQ/Orbitrap instruments; we are actively working to facilitate reading SRM data from other platforms. Search results from Mascot, Proteome Discoverer, Comet, and X!tandem can be directly imported and queried with a simple yet powerful SQLite interface based on our previously described mzResults format [14]. For example, users can filter and sort data to highlight proteins or PTMs of interest by typing simple commands at the SQLite prompt (see example queries in Supplementary Table S3 and tutorial file hosted on Github). To facilitate construction of queries, we implemented autocompletion of SQLite key words (e.g., SELECT, FROM, WHERE) as well as shortcuts for common worksheet column names (e.g., “Variable Modifications”). An integrated peptide calculator tool (PepCalc) facilitates evaluation of theoretical fragment ions (y/b for collisional activated dissociation/higher collisional energy dissociation (CAD/HCD) spectra or c/z for electron transfer dissociation (ETD) spectra) of specified charge state for spectral validation. Sequences can be adjusted on-the-fly with predicted, color-coded fragment ions remapped to the spectrum (for example, changing placement of phosphate group to validate phosphorylation site localization). For multidimensional liquid chromatography-mass spectrometry (LC-MS) studies, spectral validation can be especially laborious as it requires navigating multiple data files. mzStudio simplifies this task by allowing direct import of combined search results; associated raw data files may be loaded all at once, or cached sequentially as needed during the validation process, affording fast and seamless access across large data sets. This feature also simplifies evaluation of peak areas obtained from MS-based quantitation experiments. mzStudio can also be used to verify reporter-based quantification (TMT, iTRAQ), and supports visualization of corrected reporter intensities (i.e., corrected for reagent isotopic impurities, variation in protein input, or instrument-specific parameters such as ion injection time).

**Figure 2:**
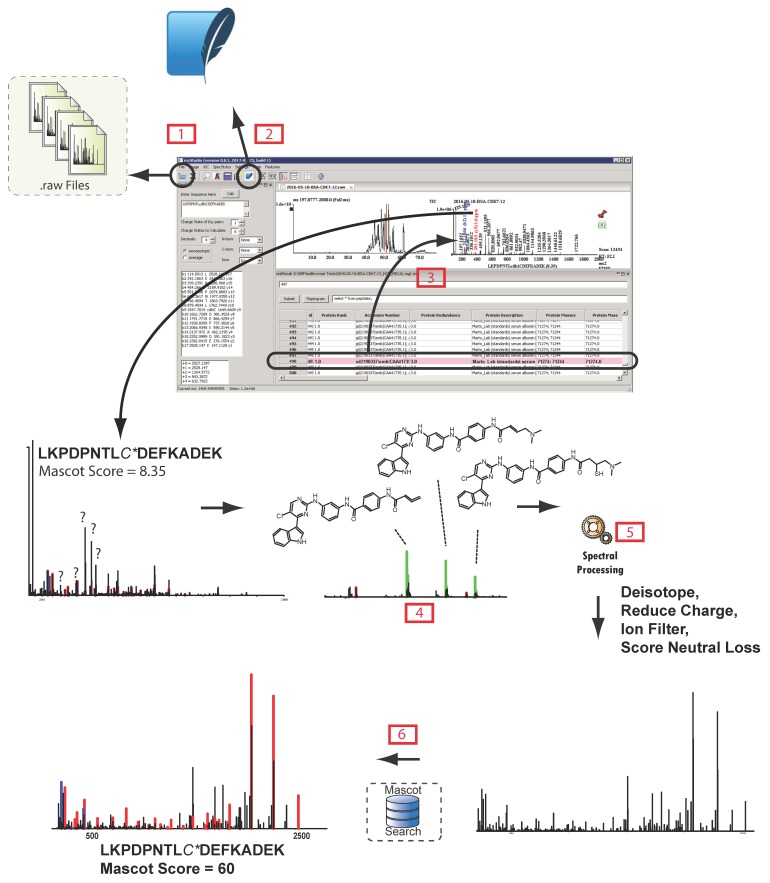
mzStudio enables custom spectral processing and direct database search of processed spectra. After opening a raw file 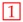 and importing search results 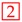, users can click individual PSMs to view annotated peaks 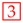. This particular MS/MS spectrum was obtained during analysis of peptides modified with the CDK7 kinase inhibitor THZ1 and yields a low-confidence Mascot score due to the presence of several inhibitor-related ions 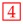 and non-canonical fragmentation pathways. Using mzStudio’s built-in spectral processing tools 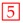, users can easily experiment with different processing algorithms (i.e., filter inhibitor related ions, reduce charge of highly charged species, and score ions from inhibitor related fragmentation pathways), and assess the impact on peptide sequence scores through the integrated search tool 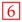.

Additional tools provide for dynamic re-evaluation of data and enable exploration of alternative hypotheses for peptide sequence, modification, or fragmentation behavior. For example, mzStudio can implement unbiased detection and visualization of MS1-based features, where each feature is an isotopic cluster over a certain time range with any associated MS/MS spectra. Once features are detected, they are directly mapped onto MS1 data. Clicking a feature tab opens a window allowing users to quickly browse to any MS1 or MS2 scan that corresponds to the feature. With this view of the data, unassigned features can be quickly identified and directly submitted for sequence assignment considering different modifications and protein databases; fragment ions assigned through each iterative search are automatically annotated within MS/MS spectra. Furthermore, mzStudio supports custom spectral processing algorithms (Supplementary Figure S1 illustrates a custom processing routine written in Python); this capability enables in-depth exploration of surprising or novel gas-phase fragmentation behavior. We used these tools to significantly improve identification rates for peptides modified with cysteine-directed covalent drugs and other chemical probes [17]. With mzStudio, researchers can add, refine, or create entirely new spectral pre-processing routines (for examples, see the example_processing_scripts folder in the Github repository), submit MS/MS data to multiple search algorithms, and assess the impact both qualitatively (improved utilization or accounting of fragment ions) and quantitatively (individual peptide score). Figure 2 illustrates a general workflow utilizing these capabilities. 

**Figure 3:**
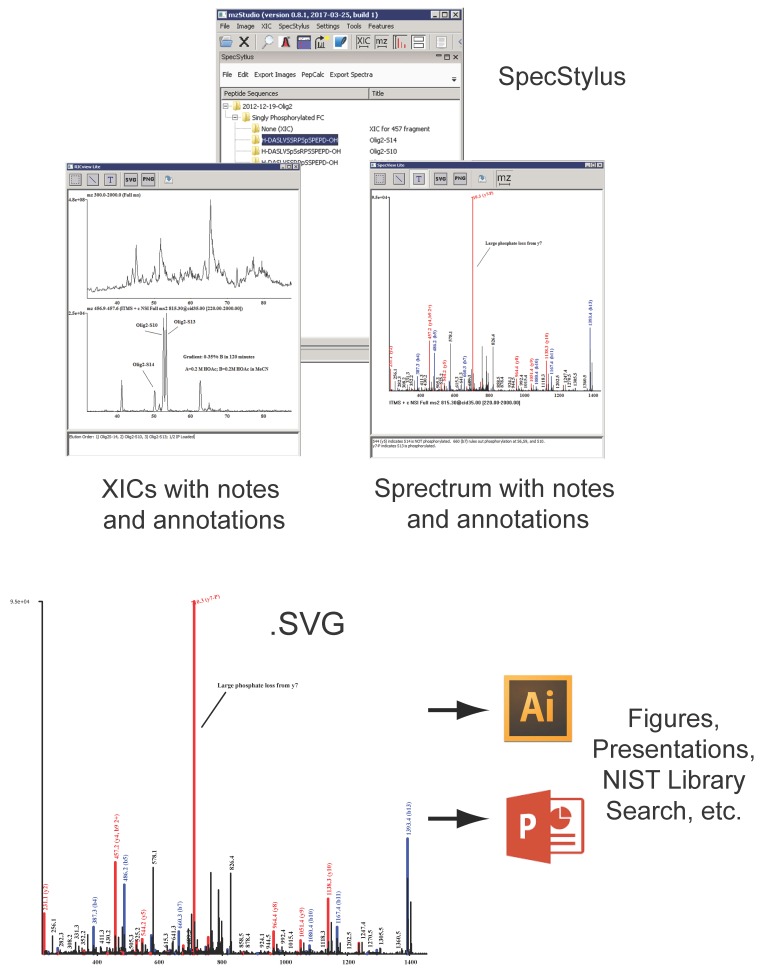
SpecStylus enables text- and graphic-annotation of mass spectra, processed spectra, extracted ion chromatograms, and search results/parameters. Collectively these figures and notes provide digital provenance for data analytic workflows and ideas supporting peptide sequence assignment. Spectra and chromatograms can be exported as .svg, .pdf, .ppt, or .png for integration into presentation material, while underlying peak lists are compatible with NIST library search tools.

It can be challenging to maintain informative, detailed records of new ideas and progress in sequence assignment when exploring novel peptide fragmentation pathways or the impact of spectral pre-processing algorithms (e.g., de-isotoping, charge-reduction, or removal of kinase inhibitor specific ions). Similarly it is difficult to test and evaluate the myriad of combinations when multiple post-translational modifications are thought to occur along a relatively short sequence of amino acids. For example, we recently utilized quantitative mass spectrometry to interrogate modifications on Olig2, a transcription factor that mediates fate choice of neural progenitor cells in the developing central nervous system and can contribute to the pathophysiology of human gliomas [18]. A set of three protein kinases works in tandem to phosphorylate Olig2 at multiple sites within the first 20 N-terminal amino acids. Indeed, these and other data [19,20] highlight the critical roles that phosphorylation on this region of Olig2 plays in its tumorigenic function. Mapping these phosphorylation sites and deciphering the kinetics to establish potential ‘priming’ phosphorylation events is an important first step in trying to identify the kinases which may represent therapeutic targets. Our work in this study required extensive analysis of MS/MS spectra to localize different and even multiple sites of phosphorylation on the same peptide fragment. To better support our work in this and similar projects, we developed the companion spectral notebook application (SpecStylus, Figure 3), which enables researchers to create a digital provenance of data analysis activities. Furthermore, spectra, processed spectra, extracted ion chromatograms, or other data projections can be annotated using an associated text box or assorted drawing widgets to catalog evidence for fragmentation pathways, phosphorylation site localization, or other spectral features. These annotations are stored in the notebook for comparison to future experiments. In addition, processing scripts, search results, and other parameters can be linked to notebook entries, thereby creating a forensic ‘chain-of-custody’ for all evidence and procedures used to support a final sequence assignment. For added convenience and portability, all intermediary steps associated with a final result can be dynamically analyzed, or further extended, independent of the original native mass spectrometry data; this feature facilitates sharing results with colleagues and assembling supplemental files for scientific journals. Finally, SpecStylus images can be exported in .png, .pdf, .svg, and .ppt format for preparation of slides or publication quality figures, while peak lists can be output as .sdb files for use with NIST library search tools.

## 3. Discussion

Data and tools derived from the human genome project are feeding efforts in mass spectrometry to quantify human proteomes in multiple biological contexts (e.g., proteogenomics). While these efforts have an abundance of scientific merit, it is also true that progress in deciphering the chemical diversity of the proteome will not be informed to a great extent by genomic data. We created mzStudio to support the detective-work that is required to carefully characterize novel modifications or surprising gas phase fragmentation behavior. Users can corroborate peptide-spectral-matches and associated quantitative measures across large, multidimensional LC-MS/MS data sets, instrument platforms, and search engines before embarking on subsequent, resource-intensive functional validation studies. Core tools provide for a feature-based analysis of data, application of custom spectral processing algorithms, and database search of processed spectra—all of which can be used to mine unassigned spectra and explore alternative hypotheses (for example, unexpected post-translational modifications). With SpecStylus mass spectra, chromatograms, scripts, and search results can be organized, documented, and annotated to provide a digital provenance of the entire landscape of evidence supporting a specific interpretation or line of inquiry. The analytic, annotation, and documenting capabilities within mzStudio will play an increasingly important role in addressing protein-level questions which are fundamentally and functionally anchored in dynamic human physiology rather than static DNA sequence.

## Supplementary Materials

The following are available online at www.mdpi.com/2227-7382/5/3/20 Figure S1: Example custom spectral processing script for mzStudio. All scripts should contain a function named “processor_function” that accepts a list of (mz, intensity) pairs (tuples). The function should return a similarly formatted processed peak list. This script performs deisotoping and charge reduction of Orbitrap HCD spectra, and removes ions related to the fragmentation of THZ1-modified peptides, Table S1: List of currently supported instrument manufacturers and platforms, Table S2: Measured times for opening files of various sizes with mzStudio using two different computers, Table S3: Exemplary SQLite queries that can be performed with mzStudio.
